# Characterization of PERV in a new conserved pig herd as potential donor animals for xenotransplantation in China

**DOI:** 10.1186/s12985-014-0212-1

**Published:** 2014-12-04

**Authors:** Fei Guo, Xiaowei Xing, Wayne J Hawthorne, Qiong Dong, Bin Ye, Juan Zhang, Qi Liang, Wei Nie, Wei Wang

**Affiliations:** Cell Transplantation and Gene Therapy Institute of Central South University, Third Xiang-Ya Hospital of Central South University, Changsha, China; Center for Medical Experiments, Third Xiang-Ya Hospital of Central South University, Changsha, China; Department of Surgery, The University of Sydney at Westmead Hospital, Westmead, NSW 2145 Australia

**Keywords:** PERV, Donor source, New herd, Recombination, Xenotransplantation

## Abstract

**Background:**

Xenotransplantation has drawn increased attention in recent years as a potential solution to the scarcity of human source donor organs. Researchers have highlighted the need to characterize the influence of porcine endogenous retroviruses (PERV) in xenotransplantation. Screening and analyzing the presence and subtype of PERV in donor source animal breeds could provide basic parameters to evaluate the biological safety of xenotransplantation from pigs to humans. We bred a new miniature porcine herd (XENO-1) after decades of investigation, the herd was purpose bred to produce a potential donor animal source for xenotransplantation. To this end we studied the animals’ PERV expression characteristics.

**Methods:**

We randomly selected 37 animals of the herd, PCR and RT-PCR based on specific primers were utilized to determine their PERV viral subtype. High fidelity PCR and restriction enzyme digestion were employed for variants detection. To thoroughly understand the PERV expression pattern, quantitative PCR was applied to measure mRNA expression levels in different tissues, At last, transfection capacity was assessed using a in vitro co-culture system.

**Results:**

Our results revealed that the XENO-1 herd was free of PERV-C and exhibited low levels of PERVs in different tissues compared to commercial pig (landrace). The XENO-1 herd showed unique variants of A/B recombination. In addition, even though there were A/B variants in the XENO-1 herd, co-culturing revealed no evidence of PERV transmission from XENO-1 tissue to human cells.

**Conclusion:**

Overall, Our results displayed an unique PERV expression pattern in a new pig herd and demonstrated its non-transfection capacity in vitro. Data in the research indicate that XENO-1 animals can serve as a better potential donor source for xenotransplantation.

## Background

World wide the shortage of human organs for transplantation has led to renewed interest in the xenotransplantation of cells, tissues, and organs. To undertake clinically effective, reliable, reproducible and safe transplants from a supply of organs that are derived from a donor animal source we must first demonstrate their suitability, efficacy and most importantly safety to reliably do this. Pigs are the most ideal donor animal because of their similar physiology to humans, large litter size, short gestational period, and genetic malleability. However, the risk of transmission of pig pathogens to humans remains a potential hurdle in pig-to-human xenotransplantation. One of the theoretically most problematic sources is the porcine endogenous retroviruses (PERV). These are considered to be a major risk because these retroviruses are integrated into the genome of all pig strains [1] and expressed in all pig cell types. Specke and colleagues demonstrated that PERV can infect both human cell lines and primary cells [2], increasing the theoretical concern of PERV infection risk in pig-to-human transplantation. There are three subtypes of PERV: PERV-A, PERV-B and PERV-C. Types A and B are human-tropic [3], whereas PERV-C is unable to infect human cells but can replicate in porcine cell lines [4,5]. However, there have been recent reports of recombinant PERV-A/C viruses that are able to infect human cells and exhibit high titer replication [6], suggesting PERV-C infectious competence. The International Xenotransplantation Association has specially established guidelines to use when transplanting pig cells in clinical trials, including the following: careful screening of the source pig herd for PERV, selection of pigs that exhibit low expression levels of PERV-A and PERV-B, and, most importantly, the selection of pigs that do not contain PERV-C [7].

Given the impossibility of eliminating PERV, the only way to eliminate or reduce the risk of potential infection is to breed specific pathogen-free (SPF) herds with low viral loads and less infectious types of PERV. To generate an ideal donor source with higher microbiology safety, we investigated for decades and bred a new pig herd XENO-1 for xenotransplantation. XENO-1 animals are interbred from Chinese Xiang and Bama pigs, their PERV-C-free ancestors were intentionally inbred more than 13 generations to breed a potential donor pig source, which easily eliminates the potential risk presented by a PERV-A/C recombinant retrovirus. The XENO-1 animals possess the characteristics of their ancestors such as smaller body size (6 month weight < 30 kg), slow-growing and early sexual maturity. Most importantly the animals are screened for designated pathogens and housed in a barrier system. To determine the characteristics of PERV subtypes and expression pattern of XENO-1, we screened for all three PERV types in peripheral blood mononuclear cells (PBMCs) from the selected 37 XENO-1 animals to confirm their lack of infection competency and showed that there was no PERV-C expression in this herd. Furthermore, we investigate the PERV expression pattern in multiple organs and tissues quantifying the viral load in different tissues relative to commercial pigs (landrace) as a basis for establishment of the utility of the XENO-1 herd.

Furthermore, a number of landmark papers have established the transmission of PERV to human cells when placed in co-culture for prolonged periods of time to mimic that of ongoing cell-to-cell contact seen if transplanted into a patient [1,2], These papers established the gold standard techniques used for proof of transmission of PERV to human cells and thus for use as a screening tool that has been widely used [8,9]. Here we utilise these same techniques to investigate the potential for the XENO-1 herd as a potential xenotransplant source.

## Results

### Sensitivity and specificity of PERV PCR primers

**Figure 1:**
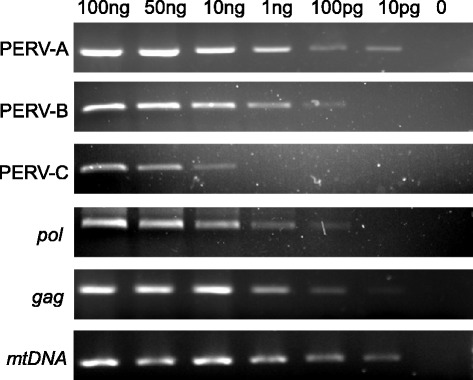
**Sensitivity and specificity of PCR for PERV proviral DNA and mtDNA, 100 ng, 50 ng, 10 ng, 1 ng, 100 pg, 10 pg and 0 indicates the varying amount of DNA templates.**

In order to demonstrate the sensitivity and specificity of our PERV PCR primers we used optimized PCR conditions and diluted the positive DNA template (PK15) samples from 100 ng to 0 pg. The limits of detection of PERV-A, *gag* and *mtDNA* were less than 10 pg, whereas the limits of detection for PERV-B and *pol* were 100 pg; PERV-C could be detected at levels as low as 10 ng DNA (Figure 1). The PCR products were single bands, suggesting that our PCR-base detection was highly sensitive and specific.

### PERV assay screen confirmed absence of PERV-C in XENO-1

**Figure 2:**
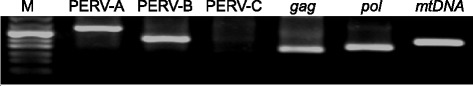
**PERV assay screen confirmed absence of PERV-C in XENO-1.** This can be seen here with the figure demonstrating PERV-A, PERV-B, gag, pol and mtDNA were detected by PCR from PBMC of xeno-1 but no PERV-C. M, DM500 marker.

Once we had confirmed the sensitivity and specificity of our PCR for detection of even extremely low-level PERV we collected blood samples from all 37-breeding core pigs. These samples were used for PERV detection using type-specific primers. PERV-A, PERV-B, *pol*, and *gag* were detected in all animals, but none of these animals had a positive PCR result for PERV-C (Figure 2). As such the herd were assumed to be PERV-C free and eliminated the possibility of the development of the potential for the A/C recombination subtype.

### Transcriptional activity of PERV

**Figure 3:**
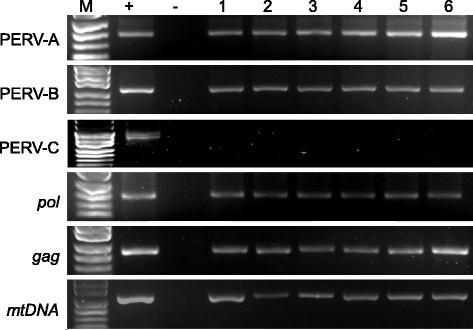
**Transcriptional activity of PERV.** Here we demonstrate PERV-A, PERV-B, gag, pol and mtDNA were detected by RT-PCR from PBMC of xeno-1 animals except PERV-C. M, 100 bp DNA ladder; +, positive control; −, negative control; lanes 1–6 indicates different pig samples.

To investigate the transcriptional activity of PERV in each animal, RT-PCR was employed using type-specific primers for all three types of PERV envelope DNA, *pol*, *gag* and *mtDNA*. The specific bands for PERV-A, PERV-B, *pol*, *gag* and *mtDNA* were detected in this assay, but no PERV-C was detected (Figure 3). DNA of PK15 as positive control was also detected in all PCR assay, but no band was found in HEK293 as negative control. These results suggest that PERV-A and B were present as demonstrated by the presence of transcriptional activity as demonstrated in these bands on the gel, however, the absence of bands for PERV-C were not present as DNA nor RNA.

### PERV expression level in different tissues

**Figure 4:**
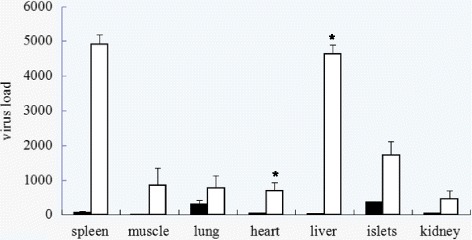
**PERV expression level in different tissues.** The expression level and comparisons are shown here with quantitative analysis results of PERV expression in different organs of xeno-1 pig 002 and a landrace pig using real-time PCR specific for PERV-pol mRNA normalized to 1 μg total RNA, student’s t-test was performed for static analysis between groups. The black bars indicate the viral load of the XENO-1 herd animal and the white bars indicate the commercial Landrace pig samples. *, *P* < 0.05.

The PERV titers in the different tissues from XENO-1 pig were much lower compared to those from the commercial pigs. The expression of PERV is characterized by the expression of full-length *pol* mRNA as previously described [10]. To fully determine the mRNA expression levels in different organs and tissues, samples from multiple organs were collected, and total RNA was isolated and quantified using real-time PCR. The highest expression of PERV in the commercial pigs was observed in the spleen and liver. Quite unlike the commercial pigs, the highest PERV expression in XENO-1 was detected in the lungs and islets (Figure 4). However, the highest viral load detected in the XENO-1 pigs was substantially lower than the highest viral load in the commercial pigs.

### Variants of PERV-A and PERV-B

**Figure 5:**
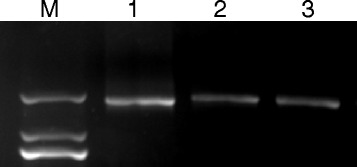
**PERV-A and PERV-B consensus PCR.** Envelope gene fragments (1.8 kb) were amplified from 3 pig samples (lanes 1–3) using consensus primers. M, DL 2000

**Figure 6:**
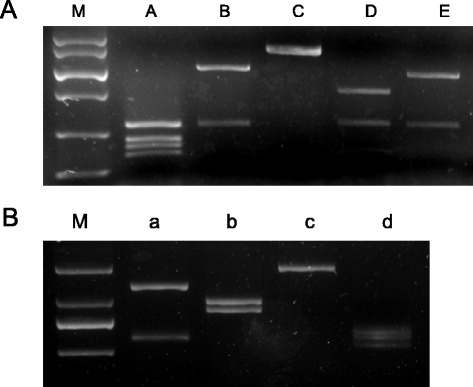
**Restriction digestion patterns of PERV clones. A**, Five patterns (A-E) of MboI digestion and **B**, four patterns (a-d) of KpnI digestion were identified in the PCR product amplified by Easy A polymerase high fidelity.

To investigate the frequency of recombination of PERV-A and PERV-B, consensus primers were used to amplify the 1.8-kb envelope gene fragment from 3 individual animals (Figure 5). The PCR products were purified using the Agarose Gel DNA Purification Kit (TaKaRa), inserted into pUCm-T vectors, and transformed into the *E.coli* strain HT115. Three hundred clones were subjected to colony PCR, and 44 positive clones were identified. The subsequent PCR products were digested using the restriction enzymes *MboI* and *KpnI.* Four *KpnI* and five *MboI* digestion patterns were identified in XENO-1 animals (Figure 6); from the two enzyme digests, we observed 7 combined patterns (Table 1). Among 44 positive clones, type B of *MboI* and type b of *KpnI* were the dominant subtypes detected (n = 32, 72.73% vs. n = 37, 84.09%). For the combined results, the primary genotypes were Bb (n = 31, 70.45%) and Aa (n = 5, 11.36%).

**Table 1:** ***KpnI*** **and** ***MboI*** **restriction digestion patterns and percentage of PERV clones**

***MboI*** **digestion pattern**	***KpnI*** **digestion pattern**	**Clone numbers**	**%**
A	a	5	11.36%
A	b	2	4.55%
B	b	31	70.45%
B	c	1	2.27%
C	b	3	6.82%
D	d	1	2.27%
E	b	1	2.27%

### Examination of PERV infectivity in co-culture

**Figure 7:**
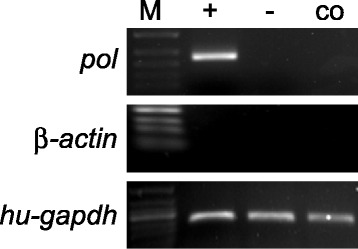
**Co-culture of XENO-1 islets and HEK293 cells.** Examination of PERV infectivity in Co-culture is demonstrated here where detection of PERV-pol mRNA in HEK-293 co-cultured with islets from xeno-1 animals by RT-PCR. M, 50 bp-DNA ladder; +, positive control, using DNA from HEK293 co-cultured with PK15; −, negative control, HEK293 cell cultured alone; co indicates HEK-293 cells co-cultured with XENO-1 islets.

In order to further define if there’s any potential for the XENO-1 animals cells to potentially transmit PERV to human cells we performed co-culture experiments. Three XENO-1 animals were sacrificed their pancreas removed and their islets isolated using a modified Riccordi method [11]. The islets were counted and the Islet Equivalent Quantity (IEQ) was calculated. Approximately 7 × 10^4^/well HEK-239 cells were seeded on the surface of 6-well plates. Then, 2 × 10^3^ islets were placed on the inserts and co-cultured with HEK-293 cells in optimized Hams F-10 medium at 37°C in a 5% CO2 incubator for 30 days. The HEK-293 cells were washed with PBS after co-culture and the mRNA was isolated. The mRNA was used to determine PERV infection by RT-PCR using pol-specific primers. Primers for porcine β-action were employed to exclude porcine cell contamination, and human GAPDH was utilized as an internal reference. Our data indicated that no porcine DNA contamination and no PERV mRNA expression occurred in the co-culture system of XENO-1 islets and HEK-293 cells (Figure 7), and we did find transmission from PK15 to HEK293 cell in the positive control assay as previously described [12]. We have therefore demonstrated there was no PERV transmission from XENO-1 cells to human cells.

## Discussion

PERV have seen resurgence in their significance as the increasing importance of xenotransplantation has been recognized. In this study, we investigated PERV in a new Chinese pig herd, these animals have been specifically inbred as potential donor animals for xenotransplantation. Here we present quite clearly verified PERV characteristics of PERV-A/B recombination and demonstrate the PERV-C deficiency of the herd and low PERV expression, furthermore, we found that there was no PERV transfection to human cells that occurred in vitro even if A/B variants existed.

Salomon and his colleagues have demonstrated PERV transmission to SCID mice transplanted with porcine islets, raising concerns about PERV transmission between species [13]. Wilson co-cultured PBMCs from the National Institutes of Health miniature pig and the Yucatan pig with several human cell lines, and found transfer and expression of PERV in all types of human cells [8], demonstrating PERV transfection competency in vitro. PERV infection of human cells in vitro suggested the presence of risks of cross-species transmission. Of all three subtypes of PERV, type C and A can be recombined as an A/C variant which possess approximately 500-fold more infectious capability than PERV-A alone [6]. However there is still no evidence of PERV infection in vivo involving pig-sourced cells or tissues. Paradis studied samples from a total of 160 subjects who had been exposed to a variety of porcine cells. The results suggested microchimerism rather than evidence of PERV transmission to human cells [14]. Patients who had been implanted with encapsulated islets were tested and found to be negative for PERV in their PBMC or plasma [15]. Although many results of studies suggest no transmission potential of infection in vivo, there are still potential theoretical latent hazards if recipients are immunocompromised, e.g., due to inflammation, fever or oncogenesis. Transmission of PERV may also be able to be activated due to immune system disorders.

In light of the increased attention on xenotransplantation, Wynyard recently presented a clinical trial from 2009 to 2014 in New Zealand [16] involving 14 recipients who received different numbers of pig islets. At 52 weeks post-transplantation, no transmission of either PERV or other porcine microorganisms were observed, further supporting the use of pig-sourced tissues and cells. Development and screening for zoonotic safe donor animals would be useful and critical to the xenotransplantation industry. Worldwide, there are many varieties of miniature pig species that possess excellent biological and physiological characteristics and have the potential to become xenotransplantation donors. However few herds have been proven to be of a standard to be deemed to be biologically and clinically safe [17]. To reduce the risk of PERV transmission, Ramsoondar and his colleagues combined nuclear transfer cloning and RNAi technology involving PERV-specific small interfering RNA (siRNA) to produce transgenic pigs that may not propagate PERV [18]. Although their results showed reduced PERV expression, the variable level of PERV expression between pigs makes it difficult to decipher whether the low viral levels are the result of siRNA alone. Additionally, some wild herds express PERV levels as low or lower than the siRNA transgenic pigs, suggesting that there may be no necessity for genetically knocking down PERV. Although there are currently no data demonstrating PERV infection in either patients or nonhuman primates receiving pig grafts, the IXA and WHO include PERV-C on a proposed list of microbiological pathogens that should be eliminated in donor source pigs to allow the use of their organs or cells for transplantation into humans. Our team screened dozens of pig breeds from the entire mainland of China over a period of several decades. From selecting appropriate progenitor stock we have inbred a new specifically designed pathogen free herd called XENO-1 that are good candidates as a source of xenotransplant donor animals. In this study, we conducted a systematic investigation of PERV expression in this herd. The XENO-1 herd was bred from intentionally screened PERV-C-free ancestors and utilizing sterile Cesarean section to eliminate all anthropozoonosis listed on the IXA pathogen-free list for donor source animals. Using DNA- and RNA- based methods we demonstrated the absence of PERV-C in all of the XENO-1 herd animals in this study (Figures 1 and 2), thus eliminating the concerns about the hazardous A/C recombination. To learn more about the PERV expression pattern, we then investigated the expression level in different tissues of one XENO-1 animal with particularly high PERV expression in PBMCs and a landrace pig that was previously described as showing low PERV expression [19]. Bittmann studied PERV mRNA expression levels by quantitative real-time PCR in multiple organs of one Yucatan pig that exhibited exceptionally high viral loads [10]. He reported that the tissues with the highest expression were the lung and spleen, whereas those with the lowest or no expression were the muscle and kidneys. In our study, the highest expression was observed in the spleen, in accordance with Bittmann, and the lowest expression was observed in the muscle, lung, heart, islets and kidney in landrace pigs. We identified a XENO-1 animal with high PERV expression in PBMCs that exhibited a unique PERV expression pattern, with high expression in the lung and islets and extremely low expression in the spleen, heart, liver, and kidney; the lowest expression was found in the muscle (Figure 4). Most importantly, XENO-1 was observed to exhibit generally low viral loads in all tissues detected, even the highest expression in the lung represented only 38% of the expression levels observed in the Landrace pigs, and there were significant differences in viral load between the XENO-1 and landrace in heart and liver (*P* < 0.05), suggesting that PERV exhibit different expression levels between herds and XENO-1 is a better potential donor animal source for xenotransplantation owing to their low PERV expression.

Next we explored variants of PERV in this herd to determine whether there was an increased probability of PERV-A/B variants due to PERV-C deficiency. Interestingly, our study showed that significantly fewer PERV-A/B variants were observed in the XENO-1 animals relative to local commercial herds previously described by Xing [20]. Using high fidelity PCR based on consensus primers, we amplified 1.8-kb segments (Figure 5). After restriction enzyme digestion, we observed only 5 *MboI* restriction digestion patterns, 4 *KpnI* restriction digestion patterns, and 7 combined patterns (Table 1) compared to 8 *MboI* patterns, 6 *KpnI* patterns, and 11 combined patterns in Ningxiang pigs. In accordance with Xing’s research, the reduction in the number of variant patterns may be due to the long duration of inbreeding and the degeneration of the viral gene. The dominant combined digestion patterns in XENO-1 were the Bb (n = 31, 70.45%) and Aa (n = 5, 11.36%) patterns, demonstrating the local characteristics of the variants and suggesting greater safety and regulation of PERV due to fewer variants.

To investigate the possibility of PERV infection in human cells, we established a transwell co-culture system to monitor the zoonotic transmission of PERV to human cells as previously described [9]. As HEK293 is consistently the most permissive human cell line for PERV infection and replication [2], we co-cultured HEK293 with XENO-1 islets that exhibited a relatively high level of expression of PERV in our study. We based this method of co-culture upon the previous landmark papers of Patience et al., Specke et al. and Jonsson et al. as they had demonstrated quite clearly that using the co-culture system they could demonstrate transmission of PERV to various human cells regardless of the use of direct cell-to-cell contact or not [1,2,9]. PERV transmission was determined by real-time PCR of the total mRNA from HEK293 cells based on *pol*-specific primers. Although Yu reported that PERV from PK15 cells could infect human cells in vitro [12], and we confirmed the transmission of PERV in HEK293 co-cultured with PK15 in our positive control landrace pig samples, we observed no significant acute effect attributable to PERV infection on the growth of HEK293 cells after extended culture with the XENO-1. Consistent with this finding, our results revealed no evidence of infection of PERV after 30 days of co-culturing (Figure 7), suggesting no direct zoonosis transmission from XENO-1 tissue to human cells.

## Conclusion

In summary, our data indicate that XENO-1, a new herd initially bred for xenotransplantation, exhibits the following desirable characteristics: no PERV-C expression, low PERV load in multiple tissues, fewer variants, and no transmission capacity. Even though there were several recombination variants in the herd, the transmission of PERV from XENO-1 tissue to human cells did not occur in vitro. Therefore, XENO-1 may represent a promising donor animal that meets the FDA industry guidelines for xenotransplantation (www.fda.gov, Guidance for Industry: Source Animal, Product, Preclinical, and Clinical Issues Concerning the Use of Xenotransplantation Products in Humans. U.S. Department of Health and Human Services, Food and Drug Administration). Our study assessed the important aspects of zoonosis using a novel herd of potential donor source animals validating this herd’s ideal characteristics. A more thorough understanding of the latent influence of PERV on XENO-1 animals and human cells precipitates further study, including sequencing analyses of variant patterns and phenotypes, as well as functional studies of PERV-infected cells.

## Materials and methods

### Animal ethics

All research was undertaken following approval and in accordance with the recommendations of the Animal Research Committee of Central South University. Pigs were provided by Hunan Xeno Life Science (Changsha, China) and housed in the Experimental Animal Facility of Central South University (Changsha, China).

### Animals

Following more than a decade of intense searching the XENO-1 herd has been interbred from two different conserved Chinese native pig herds that were identified by screening over 20 herds of pigs throughout China; they are housed in the DPF Animal Facility of Hunan Xeno Life Science (Changsha, China). There are now 115 animals as the core of the herd. The commercial outbred (Landrace) animal used for comparison were supplied by the Experimental Animal Facility of Central South University.

### Isolation of PBMC

Peripheral blood samples from 37 individual pigs (age 4–8 month, 17 male and 20 female) were randomly collected into tubes containing heparin. PBMCs were extracted as previously described [21].

### Reagents and cells

PK15 and HEK-293 cells were provided by cell center in Central South University (Changsha, China). Genomic DNA extraction from all cells was performed using a DNA isolation kit (Tiangen, Beijing, China) per the manufacturer’s instructions. Total RNA from different tissues and cells was extracted according to the manufacturer’s instructions (Qiagen, Valencia, CA). The DNA and RNA concentrations were measured with ultraviolet (UV) spectrophotometry. Reverse transcription was performed using the Super ScriptTMIII first-strand synthesis kit (Invitrogen, Carlsbad, CA, USA). Primers were synthesized by Sangon biotech (Sangon, Shanghai, China).

### Detection of PERV proviral DNA and RNA by PCR and RT-PCR in PBMCs from pigs

The PERV genes *pol* and *gag*, porcine mitochondrial (mt) DNA that codes cytochrome oxidase subunit II (*CoII*), and all three types of PERV envelope-coding DNA (PERV-A, PERV-B and PERV-C) were detected by PCR and RT-PCR using specific primers (Table 2). DNA and RNA of PK15 were used as positive controls, HEK293 cells were employed as negative controls. The PCR reaction volume (20 μL) contained the following: 12 μL nuclease-free water, 2 μL 10× PCR reaction buffer, 1.6 μL 25 mmol/L MgCl_2_, 2 μL 2.5 mmol/L dNTP, 0.4 μL Taq DNA polymerase (Takara, Dalian, China), 0.5 μL primer mix and 1.5 μL DNA. The cycling reaction was performed in a programmable thermal cycler (PE9700) under the following conditions: denaturation at 95°C for 150 seconds; 35 cycles at 94°C for 40 seconds, 58-60°C for 40 seconds, and 72°C for 40 seconds; and extension for 5 minutes. The PCR products were then run on a 2% agarose gel. The PCR sensitivities for PERV and *mtDNA* are also described here using a serial dilution of a positive sample (Figure 1). All PCR assays were repeated at least once to confirm the results.

**Table 2:** **Primers for PCR and RT-PCR amplification of the different PERV subtypes,** ***mtDNA*** **, pocine** ***β-actin*** **and human GAPDH**

**Gene**	**Primer sequence**	**Fragment size (bp)**
PERV-A	F: 5′-TGGAAAGATTGGCAACAGCG-3′	360
	R: 5′-AGTGATGTTAGGCTCAGTGG-3′	
PERV-B	F: 5′-TTCTCCTTTGTCAATTCCGG-3′	264
	R: 5′-TACTTTATCGGGTCCCACTG-3′	
PERV-C	F: 5′-CTGACCTGGATTAGAACTGG-3′	281
	R: 5′-ATGTTAGAGGATGGTCCTGG-3′	
*pol*	F: 5′-CCACAGGGCAACGGCAGTATCC-3′	212
	R: 5′-TTGGAGGGTCAACACAGTGATGG-3′	
*gag*	F: 5′-CGGCAAGAGAAGAATTTGACT-3′	188
	R: 5′-CAGTTCCTTGCCCAGTGTCC-3′	
*mtDNA*	F: 5′-TCACCCATCATAGAAGAACTCCTACA-3′	281
	R: 5′-TTTTACGGTTAAGGCTGGGTTATTAAT-3′	
*β-actin*	F: 5′-CACGCCATCCTGCGTCTGGA-3′	100
	R: 5′-AGCACCGTGTTGGCGTAGAG-3′	
GAPDH	F: 5′-CAAGGTCATCCATGACAACTTTG-3′	496
	R: 5′-GTCCACCACCCTGTTGCTGTAG-3′	

### PERV mRNA expression in different tissues relative to a commercial pig

One XENO-1 animal (female, 5 months old, No.M002) in which elevated PERV expression was detected and one Changsha native commercial pig (landrace, male, 5 months old) were sacrificed, Samples were taken from various organs and analyzed for PERV expression. All tissues were immediately removed and perfused with PBS (Sigma, MO, USA) to remove any free blood from the tissue. To determine the mRNA expression levels, total RNA was isolated, and RT-PCR was performed using 1 μg RNA from each organ tissue. Quantitative real-time PCR was performed to detect the load of PERV in the XENO-1 and the land race pigs relative to *β-actin*. Real-time PCR was based on primers specific for the *pol* sequence using the Invitrogen SuperScript III platinum system, the MX3000 thermocycler (Stratagene), and primers specific for the PERV gene *pol* and porcine *β-actin* (Table 2). Results of three independent experiments were averaged to determine the relative quantification. All results are expressed as mean values ± standard deviation (SD). Comparisons between groups were performed by Student’s *t*-tests. All comparisons are 2 tailed, using SPSS10.0. *P* < 0.05 was considered significant.

### Analysis of PERV env-A and env-B variants

Easy-A High-Fidelity Taq DNA polymerase (Stratagene, CA, USA) was used to amplify the 1.8-kb envelope gene fragments from three XENO-1 individuals using consensus primers for both type A and type B viruses. The consensus primers (sense primer: 5′-CATGCATCCCACGTTAAGC-3′, antisense primer: 5′- ACCATCCTTCAAACCACCC-3′), which were chosen from the highly conserved regions at either end of the PERV-A and PERV-B envelope genes, were used to search for novel variants. PCR products were primarily checked by electrophoresis. Approximately 1.8-kb PCR fragments were purified using a DNA purification kit (Takara, Dalian, China) and mixed. These fragments were inserted into pUCm-T (Tiangen, Beijing, China) vectors and transformed into the *E. coli* strain HT-115 (Tiangen, Beijing, China). Three hundred single positive clones were picked for PCR amplification, and the PCR products were analyzed using the restriction enzymes *KpnI* and *MboI* (New England Biolabs, MA, USA).

### Porcine islet and HEK293 cell co-culturing to monitor PERV transmission

Islets from three XENO-1s were freshly isolated as previously described [22] and co-cultured with human embryonic kidney (HEK) 293 cells as also previously described [9]. Due to the difficulty in culturing islet cells long-term we utilised the culture media based upon the improved method [23] in Hams F-10 medium (Gibco-Invitrogen, Grand Island, NY, USA) containing 10% fetal bovine serum, 10 mM glucose, 50 mM isobutylmethlxanthine (Sigma-Aldrich, St. Louis, MO, USA), 2 mM L-glutamine (Invitrogen, Carlsbad, CA, USA), 10 mM nicotinamide (Sigma-Aldrich, St. Louis, MO, USA), 100 U/ml penicillin and 100 ug/ml streptomycin (Invitrogen, Carlsbad, CA, USA), CaCl_2_ 0.236 g/L, Hepes 80 mM, NaHCO_3_ 21.3% (Sigma-Aldrich, St. Louis, MO, USA), with full media changes per protocol. The cells were co-cultured for one month at 37°C and 5% CO_2_ to estimate the potential retroviral transmission capacity of low PERV-bearing xenografts. Long-term co-culture assays were performed in 6-well tissue culture plates with inserts (Transwell®, Corning Inc, NY). This is currently the gold standard technique used as described by Jonsson [9], the system employs a membrane with 0.4-μm diameter pores, which keeps the two cell types physically separated but allows the diffusion of nutrients and small molecules, including virus particles approximately 0.1 μm in size (including PERV). Approximately 7 × 10^4^ HEK293 cells were cultured on the surface of 6-well plates whereas 2 × 10^3^ IEQ islets were initially co-cultured on top of the insert membranes. 7 × 10^4^ PK15 (insert) were also co-cultured with 7 × 10^4^ HEK293 (well) as positive control, HEK293 were cultured alone as negative controls. Every 72 hours the HEK293 cells were subjected to mild trypsinization and diluted into 3 parts fresh medium, excess 293 cells were used for genomic DNA preparation. At one-month intervals, the islets were removed by removing the inserts. The HEK293 cells were washed twice with PBS and subsequently subjected to real-time PCR for PERV detection. The human GAPDH gene was used as an internal reference, porcine *β-actin* was employed for porcine cell contamination. Three independent experiments were employed to determine the transmission.

## Abbreviations

PERV: Porcine endogenous retroviruses
IEQ: Islet equivalent quantity
